# Biodiversity into your hands - A call for a virtual global natural history ‘metacollection’

**DOI:** 10.1186/1742-9994-10-55

**Published:** 2013-09-17

**Authors:** Michael Balke, Stefan Schmidt, Axel Hausmann, Emmanuel FA Toussaint, Johannes Bergsten, Matthew Buffington, Christoph L Häuser, Alexander Kroupa, Gregor Hagedorn, Alexander Riedel, Andrew Polaszek, Rosichon Ubaidillah, Lars Krogmann, Andreas Zwick, Martin Fikáček, Jiří Hájek, Mariano C Michat, Christopher Dietrich, John La Salle, Beth Mantle, Peter KL Ng, Donald Hobern

**Affiliations:** 1 Zoologische Staatssammlung, Münchhausenstr. 21, 81247 Munich, Germany; 2 GeoBio Center, Ludwig-Maximilians-University, Munich, Germany; 3 Swedish Museum of Natural History, SE 104 05, Stockholm, Sweden; 4 Systematic Entomology Lab, USDA-ARS, c/o NMNH, Smithsonian Institution, Washington DC, USA; 5 Museum für Naturkunde, Invalidenstr. 43, 10115 Berlin, Germany; 6 Staatliches Museum für Naturkunde Karlsruhe, Erbprinzenstr. 13, 76133 Karlsruhe, Germany; 7 Natural History Museum London, Cromwell Road, London SW7 5BD England; 8 LIPI Division of Zoology (Museum Zoologicum Bogoriense), Cibinong, West Java, Indonesia; 9 Staatliches Museum für Naturkunde Stuttgart, Rosenstein 1, 70191 Stuttgart, Germany; 10 Department of Entomology, National Museum, Kunratice 1, 148 00 Praha 4, Czech Republic; 11 CONICET - Laboratorio de Entomologiá, Departamento de Biodiversidad y Biologá Experimental, Universidad de Buenos Aires, Av. Int. Guïraldes s/n, Ciudad Universitaria, C1428EHA Buenos Aires, Argentina; 12 Illinois Natural History Survey, University of Illinois, 1816 S Oak St., Champaign, IL 61801, USA; 13 Atlas of Living Australia, CSIRO Ecosystem Sciences, GPO Box 1700, Canberra, ACT, 2601, Australia; 14 Australian National Insect Collection, CSIRO Ecosystem Sciences, GPO Box 1700, Canberra, ACT 2601, Australia; 15 National University of Singapore and Raffles Museum of Biodiversity Research, Department of Biological Science, National University of Singapore, 14 Science Drive 4, 117543 Singapore, Singapore; 16 GBIF Secretariat, Universitetsparken 15, 2100 Copenhagen, Denmark

**Keywords:** Mass digitization, Natural history collections, Collection access, Metacollection, Cybertaxonomy, Online resources, Robotic imaging, Accessions, DNA extraction vouchers

## Abstract

**Background:**

Many scientific disciplines rely on correct taxon delineations and identifications. So does a great part of the general public as well as decision makers. Researchers, students and enthusiastic amateurs often feel frustrated because information about species remains scattered, difficult to access, or difficult to decipher. Together, this affects almost anyone who wishes to identify species or verify identifications. Many remedies have been proposed, but we argue that the role of natural history collections remains insufficiently appreciated. We suggest using state-of-the-art mass imaging technology and to join forces to create a global natural history metacollection on the internet, providing access to the morphology of tens of millions of specimens and making them available for automated digital image analysis.

**Discussion:**

Robotic high-resolution imaging technology and fast (high performance) computer-based image stitching make it now feasible to digitize entire collection drawers typically used for arthropod collections, or trays or containers used for other objects. Resolutions of 500 megapixels and much higher are already utilized to capture the contents of 40x50 cm collection drawers, providing amazing detail of specimens. Flanked by metadata entry, this helps to create access to tens of thousands of specimens in days. By setting priorities and combining the holdings of the most comprehensive collections for certain taxa, drawer digitizing offers the unique opportunity to create a global, virtual metacollection.

The taxonomic and geographic coverage of such a collection could never be achieved by a single institution or individual. We argue that by joining forces, many new impulses will emerge for systematic biology, related fields and understanding of biodiversity in general.

Digitizing drawers containing unidentified, little-curated specimens is a contribution towards the beginning of a new era of online curation. It also will help taxonomists and curators to discover and process the millions of “gems” of undescribed species hidden in museum accessions.

**Summary:**

Our proposal suggests creating virtual, high-resolution image resources that will, for the first time in history, provide access for expert scientists as well as students and the general public to the enormous wealth of the world’s natural history collections. We foresee that this will contribute to a better understanding, appreciation and increased use of biodiversity resources and the natural history collections serving this cause.

## Introduction

Species are the currency of comparative biology. Scientists from many biological disciplines, including community ecology, conservation biology, pest management, biosecurity and biological control rely on scientifically sound, objective species data, often also on other taxonomic ranks. However, large-scale identifications, i.e. the identification of large numbers of specimens for specific, often project-related purposes, are often not feasible. Researchers, students, parataxonomists, and enthusiastic amateurs often feel frustrated because information about species remains scattered, difficult to access, or difficult to decipher (e.g. available only in highly technical jargon or non-native languages).

Several proposals have been put forward to remedy this situation: moving taxonomic revisions [1] and printed sources [2] into cyberspace; establishing official authority files of taxonomic names [3], including universal registries for these [4,5]; comprehensive species- and population-level DNA barcode databases [6,7]; databases of occurrence data [8]; online communities using image databases for identification and research [9,10], data portals including species-pages and associated resources [11-14]; collaborative data publishing frameworks [15]; interactive online identification keys [16]. Each of these approaches addresses some aspect of the problem and they increasingly interact and complement each other. For example, species occurrence data from diverse data providers including Global Biodiversity Information Facility (GBIF) are error-checked and mapped onto Discover Life species pages and the maps provided to the Encyclopedia of Life (EOL). The journal ZooKeys [17,18] simultaneously publishes all taxonomic acts in the journal as well as in a versioned wiki format which allows subsequent addition of locality data or ecological information on the species [19]. Botanists are in the process of compiling a global resource documenting all plant types [20]. This Global Plants Initiative (GPI) funded by the Mellon Foundation created a partnership of more than 190 museums and herbaria from more than 60 countries and illustrates well how digitization efforts could turn global. GPI uses Journal Store (JSTOR) plant science [21] as its data portal, interlinking with resources from JSTOR digitized literature resources as well the Biodiversity Heritage Library (BHL).

Anyone with the need for accurate and verified species identifications, be they a researcher at a museum in Madrid, a student at a university in Sumatra, a parataxonomist at a community ecology research center in Papua New Guinea, or an amateur entomologist in England, would at some stage want to experience feedback on identifications. A virtual community approach to providing such feedback would improve species identifications by principal experts and beyond the coarser “morphospecies” or generic identifications commonly applied in community ecology. For most of the better known species multiple taxon pages are already online (e.g., Discover Life alone serves 1,226,003 species pages), many of which feature digital images, maps, and scanned text from revisions, that facilitate making and verifying species identifications. At sites such as Discover Life, dynamic identification guides linked to these species pages and derived in part from the same content (e.g., images) allow even non-experts to achieve efficient and reliable species identifications. Other interactive portals, such as Project Noah [9] and iNaturalist [10] allow the submission of geocoded photographs for identification by members of a wider community.

We will focus here on the role of natural history collections and suggest that mass digitization of collection holdings in the form of high-resolution images, including whole drawer digitization, is the way forward and will provide a better, faster and more democratic access to collections and biodiversity than ever before - essentially to put biodiversity in your hands.

## Discussion

We argue that natural history collections are the largest and most important source of authoritative biodiversity data (for research but also web-based initiatives such as GBIF). They provide, in many cases, our only insight into historical trends of critical importance for conserving resources in an era of global change. However, most of the material in museum collections remains undiscoverable, with many important specimens not available to the research community. This situation is especially true for arthropods, which constitute the vast majority of named organism diversity [22]: Collections continue to represent the most important resources for arthropod species discovery and identification.

Species pages and other virtual resources are of great and increasing value. They ultimately rely upon well-curated, specialized collections as the source of their most extensive and reliable data, and ongoing reciprocal feedback between collection users (professional and amateur taxonomists) and online data portals is the most effective way to optimize data quality. The most relevant specimen collections are those housed in the natural history museums around the world. With about 3 billion specimens, accumulated over 250 years, these are the primary archives and physical databases of global species diversity [23] and serve as evidence and foundation for all downstream applications including print publications and web resources. For researchers, visiting such an institution is usually the best option to achieve scientifically sound identification for species where no modern identification tools are available. However, travel is expensive, and visa regulations may prevent many research visits. Moreover, holdings vary considerably across collections, with only very few having both broad taxonomic and geographic coverage (such as the Natural History Museum in London, the American Museum of Natural History in New York, and the Smithsonian Institution in Washington, DC). However, there are many other institutions and individuals with excellent, specialized collections scattered across the globe. Expert taxonomists will usually spend years visiting a number of relevant collections, or request loans from others, but many researchers and amateurs will even find it impossible to travel so extensively. Given time and funding constraints, and increasing difficulties for many host institutions to supply loans, it is impractical for a researcher to visit or request specimen loans from every collection that may conceivably house specimens in the taxon of interest. Thus, researchers often focus their attention on the larger collections while institutions with more modest holdings are either overlooked or intentionally ignored.

Furthermore, important specimens go undiscovered for many years, despite the best efforts of the curators and technical staff, because large collections often do not have exhaustive inventories.

Digitization efforts in botany in general benefit from the method of preservation of herbarium specimens - many samples are dry on virtually two-dimensional sheets that contain the specimen(s) as well as printed or written documentation. These can readily be scanned and botanists are therefore very advanced with digitizing their collections. Apart from the GPI, there are other large-scale virtual herbarium initiatives, for example the Australian Virtual Herbarium and the US Virtual Herbarium (USVH) project, the latter aiming to digitize (database, image and georeference) all specimens in all herbaria in the United States [24,25]. However, if we are to create a truly comprehensive online collection coverage of global diversity, is the main challenge is to deliver a fundamental change in the digitization of zoological collections. While the digitization of types and selected individual specimens is feasible, this may e.g. not be true for larger series of individual insects on a one-by-one basis. Existing collections house many millions of such specimens and are constantly growing. Traditional digitization methods usually require handling of individual specimens, a process that is not only time consuming but also comes with substantial risk of damage to many specimens. Technological developments as well as a change in the digitization paradigm create the launching pad for truly accelerated opening of collection holdings. A special issue of ZooKeys sheds light on recent and ongoing approaches from large natural history collections and universities around the globe [26].

Several national or global initiatives are already actively engaged in aggregating, managing, exposing and sharing digital information from natural history collections, including GBIF [8], Encyclopedia of Life [11], Atlas of Living Australia [27], and the US Virtual Herbarium [24]. It is now time for these initiatives to work together to create a comprehensive global virtual online metacollection covering most of the Earth’s species diversity using state-of the art digital imaging technology. Here, we will focus on drawer- or tray-based collections (or in technical terms, “container-based”). Typical examples of such collections are arthropods, mollusk shells, as well as paleontological specimens, birds, eggs, and many other types of specimens. Although of course and certainly not always feasible, we argue that in many taxa, body size and characters visible in dorsal view do provide a wealth of information, even allowing identifications to be made or verified. Many arthropods, including but not limited to larger species that are well known to amateurs, are readily identified to species by inspecting them on high resolution images in dorsal view. Equally important would be the ability to rule out the possibility of a specimen belonging to a given species, as in the example of a quarantine worker who might be concerned about the presence of a key pest. Access to comprehensive overviews of the range of variation in species or a genus will help to address an increasing risk on the Internet, that observers are unaware of the existence of multiple broadly-similar species and naïvely copy identifications from other web observations.

However, initial identifications using these images are only the beginning. High-resolution images can sometimes provide clear views of the label data associated with a pinned insect specimen, in which case systematists may be able to locate lost type series and new collecting localities or dates. Images can also assist a researcher in determining whether type specimens actually have to be borrowed, or selecting which specimens should be loaned from a museum for further study of characters that cannot be assessed on the image. Drawer images can help a researcher decide where photography of particular specimens from different perspectives might help. A scientist interested in the evolution of colour pattern, or wing shape, could use drawer images to infer infraspecific variation and polymorphisms deduced from specimen series, and interspecific variation by comparing different species. Knowledge of such variation is widely available for frequent species, in which individual collections hold entire series, but lacking for rare species, where the only available specimens may be distributed over many collections. Similarly, it has been suggested that fluctuating asymmetry in insect wings could be an indicator of environmental stress [28].

These images would enable better planning prior to an actual visit to a collection, allowing remote access to a collection to answer questions like “how many species X do you have from locality Y?” Drawer images show how many specimens exist and where they come from (when label data are visible or annotations/metadata were entered after scanning) - enabling ecologists and biogeographers to specifically request additional metadata. Ultimately, the everyday utility of biodiversity science could be similar to large scale geographical digitization efforts like Google Earth, Maps or Streetview, which are used by millions of people every day to plan their lifes.

**Figure 1:**
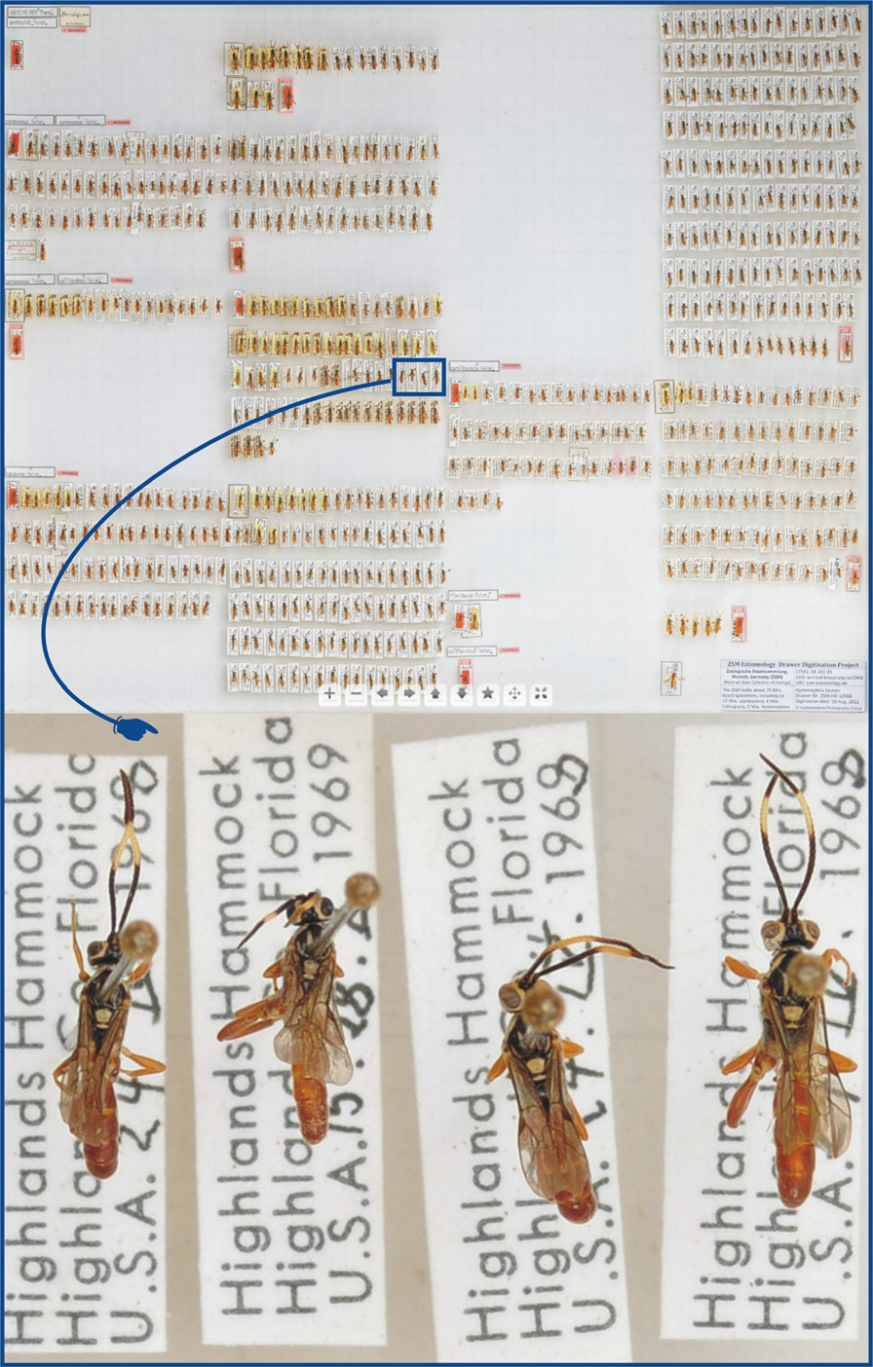
A 300 MP scan of an Ichneumonidae drawer (ZSM Entomology digitization group 2012), and a magnified view of some specimens.

Existing drawer digitization systems such as SatScan [29-31], DScan [32,33] or GigaPan [34,35] are capable of rapidly digitizing an unprecedented number of specimens and species with an image resolution that allows, in many cases, identifications to family, genus, and sometimes even species level (Figure 1). This single-handedly will revolutionize the way natural history collections can reach out to the general public. For example, the DScan prototype that is used at the Zoologische Staatssammlung in Munich takes about eight minutes to capture a 41 × 52 cm sized insect drawer at a stunning 500 megapixel resolution [32,33] - and much higher resolutions are already feasible on the hardware and storage side (see also [30]). The Gigapan System, as currently used at North Carolina State University and the National Museum of Natural History (USNM, Washington DC), can capture, stitch, and upload to the web 200 megapixel images (created from 30 raw images) of a single drawer of insects in under three minutes, with an additional 3–5 minutes for species annotation on the resulting image [36]. Several stakeholders are currently pursuing mass digitization efforts, for example the Natural History Museums in London, Berlin, Leiden, Munich, Washington DC, North Carolina State University, the Australian National Insect Collection in Canberra, and the USNM [30,31,37,38]. The US National Science Foundation (NSF) has recently allocated several million US$ for mass digitization projects and for the development of a cybertaxonomy infrastructure - explicitly aiming at creating resources for ecologists (INVERTNET [39]; SCAN: [40]). INVERTNET suggests taking drawer imaging from 2D to 3D scanning, as it is technically feasible to automatically manipulate and digitize a drawer to produce a partial 3D reconstruction of specimens.

Drawer digitization does not merely provide photographic depictions of species, but delivers extra value – namely the *context of a specie*s among its congeneric species and the context of an individual among conspecific individuals.

Once drawers have been digitized at high resolution, unprecedented possibilities exist. Image cropping will deliver the material for individual species pages where needed. Cybercuration allows for the creation of a new, virtual collection: species from collection ‘A’ can be combined with species from collection ‘B’, and so forth, producing a unified global virtual metacollection. Ideally, this would include the holotype for each species, helping to detect misidentified holdings and avoid circulation of such misidentifications on the web which is not desireable [41]. By pooling their information, every ‘real’ or ‘analog’ museum or other collection would contribute to one virtual global biodiversity metacollection, which would be far more complete than individual collections will ever be. Museums of countries that contain biodiversity hotspots, such as Indonesia, would have the opportunity to make their collections available and provide research incentives for several biological disciplines, including taxonomists, entomologists, and ecologists. This virtual collection could be linked to and provide illustrations for existing biodiversity data portals (Encyclopedia of Life (EOL), Discover Life, Atlas of Living Australia, etc.). It will be this *interaction* that makes the final product comprehensive. Ultimately, cropped images of a series of specimens belonging to the same species in one collection could be combined with images of the same species from other institutions, thus allowing simultaneous comparison of insect specimens from several or even dozens of participating museums. This will provide a comprehensive virtual ‘metacollection’ of a species that would otherwise be hard to achieve. Most importantly, the foundation for this type of comparison, i.e. specimen drawer images, can be created within a relatively short time frame.

A virtual collection can never replace a real collection of the physical specimens. The collection object will remain the primary reference for biological diversity where all facets (e.g. complex morphology and fine structure, anatomy, genetic material, chemical compounds, pollen on insects or stomach contents, to name but a few) are required. It also does not replace expertly conducted specimen databasing, particularly when such efforts are incorporated into the overall workflow of a revisionary taxonomic project that includes comparative morphological study as well as imaging and other data capture.

As a meaningful strategy, it seems advisable to initially focus on key taxa to satisfy demand from ongoing or scheduled research initiatives that would benefit from the proposed virtual collection. Demand-driven prioritization will focus the available resources on those questions where taxonomic or ecological research is most promising. This might include neglected taxa, geographical regions rich in biodiversity but with insufficient taxonomic infrastructure, or ecological question such as organism interactions (e. g. pollination or herbivores). A comprehensive virtual collection will easily allow researchers to re-arrange species according to multiple criteria and corresponding research questions, e.g. arrangement according to the latest phylogenetic reconstructions, according to their lifeform, habitat type, behavioral data, updated classifications, geography, host plants, parasitoid complexes, and plant communities. In contrast, all physical collection allow the organization only by a single, usually taxonomic research criterion.

Furthermore, drawer digitization will uncover morphological inconsistencies within a species, within and across collections, which could be due to misidentifications and phenotypic differences in size and/or color due to geographical variation; and possibly drastic divergence in identification quality, labeling practices, and updating of nomenclature that might stimulate revision of curatorial practice. In addition, by using planar telecentric lenses, images can be created with minimal distortion, which enables accurate measurements of specimens anywhere in the image [30,31]. This will allow for morphometric data analyses of virtual online collections of species derived from multiple collections without a single loan or museum visit.

Drawer images open a plethora of opportunities to extract information not only by manual inspection by humans, but also by extracting information about species and specimens using image analysis and feature extraction software [34]. Depending on the taxon and the size of the label, locality labels are often partially visible from above and can be read by optical character recognition [36,42,43]. With partial 3D scanning, where photos are not only taken vertically from above a drawer but also from angles, typically more of the label becomes readable [44]. OCR-techniques struggle with some types of labels, e.g. handwritten labels, and in such cases crowdsourcing approaches seem promising for large scale transcription of metadata [45,46]. Labels are added to each specimen after mounting, and specimens accumulate other labels as they are curated, revised, databased, measured, DNA extracted etc. A dedicated label with a machine readable code (such as a barcode or QR code) that is readable on a drawer image will let technicians associate each specimen in a collection with a globally unique identifier, e.g. by employing a HTTP URIs. These serve both as a stable persistent identifier for the specimens and as mechanism in the Linked Open Data Cloud to access more information about the specimen. Machine readable codes can be automatically extracted from drawer images and facilitate the ability of the scientific community to track individual specimens in a collection and across collections (e.g. type specimens, or specimens used in a publication). This resource will enable the direct global access to a particular specimen. Similarly, registered users could tag or annotate specimens or drawers, very similar to websites such as Flickr [47], thus increasing the information content associated with any given specimen. A simplified approach would be inclusion of one machine readable code in each unit tray or beside the taxon label in non-tray based drawers. Information encoded could be a link to a specimen database, species page in the internet containing a huge variety of species related data such as ecology, Genbank accessions, taxonomic description, references, detailed digital images and so forth.

On top of these processes that are focused on collection management and specimen metadata, in many cases automated image analysis can be used. Methods developed for the identification of carefully prepared individuals, e.g. based on wing venation or color patterns [48] need to be adapted to work on the drawer (container) level. Although such systems will not be perfect, they will allow to detect useful indications of unexpected variation within neighboring specimens. Supported by a new system for the detection of species grouping within a container (e.g. in insects, specimens of one species are usually separated by some extra space from the specimens of another species) this may lead to the detection of misidentifications and ultimately the detection of overlooked new species, the hidden treasures of collections.

### Light into the darkest corners

One of the hidden values of every natural history collection is their drawers filled with unsorted and unidentified specimens. The unsorted material of all natural history collections combined contains a wealth of new country, province, and state occurrence records. In addition it is expected to contain a significant amount of new, yet undescribed species. With a virtual global collection, researchers will have the chance to screen this material rapidly and online and ask for specific loans. In addition, they and qualified amateurs could provide generic or species identifications for specimens within their range of expertise, thus providing an *online curation* of natural history collections, including those unable to support resident specialists for the taxa in question (e.g., [34,49]).

### Practical limitation 1: what about metadata?

An often expressed concern with respect to collection mass digitization is: where are the metadata? For example, the images do not easily conform to GBIF data standards, and often labels are not fully visible from above. From a curatorial point of view, the problem is simple: human resources are extremely sparse, and entering millions of specimen records is not a realistic short-term priority for many institutions and taxa, though it clearly should be. Therefore, data entry has to be focused on those groups where data availability is currently required - for example, bees might be of greater interest currently than central Asian rove beetles.

Entering proper specimen data often requires highly trained staff capable of deciphering old labels and georeferencing ambiguous localities. For such an undertaking, specimens usually need to be handled, which is not desirable from a curatorial point of view, because potential damage to specimens has to be avoided. Smith & Blagoderov [26] estimated that “approximately 90% of the time required for digitization is spent on capturing metadata and labelling specimens”. We suggest that by mass digitizing collections, we provide a window into what is in a collection in the first place, and attached metadata might be sparse initially. It is technologically feasible to attach basic data, stored within the image itself, to each specimen, or clusters of specimens, during the image processing step [31, 34, and others in 50]. Once researchers or other stakeholders require more data, “metadata entry on demand” can be requested, or more elaborate data can be entered as a priority activity or as databasing funding becomes available for certain taxa. These metadata can be tagged to the specimens and linked with a database (e.g. GBIF; discussed further below). Thus, while drawer digitization does not replace metadata entry, it can rapidly provide a great deal of rich information until such time as complete metadata has been captured. Services such as GBIF should be extended to facilitate discovery of such partially curated material alongside more complete data records. For large scale transcription of metadata, some projects have developed successful crowd sourcing approaches where volunteers are engaged as citizen scientists [45,46]. We have suggested above that inclusion of a machine readable label with a unique specimen identifier for each species could link the drawer image and specimen data database.

### Practical limitation 2: the taxon name labels are not visible from above

Many collections use a unit tray system where each species has its own (or several) tray(s) within the collection drawers. Often, the label with the taxon name is not attached to the bottom of the tray, but vertically against one of the tray walls. Such labels cannot clearly be read from above, and in cases such as these, each tray would need to be annotated electronically after generation of the final drawer image. But this is technically feasible at the metadata collection step as described above, where staff would enter the taxon name, and if possible its unique identifier, a link to a museum database, Zoobank, Wikispecies *et cetera*. Alternatively, vertical header labels can be replaced with horizontal labels as part of the imaging workflow. Another option are ‘snapshots’ available through Gigapan, where species names or other information can be assigned by the user to any given region of interest within a drawer; within the snapshot, a link can be inserted that reaches another resource such as EOL or Genbank. Such windows will also link to Zoobank and contain identifiers of species, author names, and so forth. 3D scanning mentioned above might remedy this problem, allowing virtual online tilting of drawers to reveal the vertical taxon label at the front of unit trays [50].

New technical systems can be developed, where a set of cameras (one perpendicular, and four at 45° angle around it focusing on the same center) are used instead of one. In mapping, new photogrammetry uses similar techniques (albeit looking outwards from the airplane) and software to switch from perpendicular view to side-view is available e.g. for Microsoft and Google maps. Having 45° angled pictures from four sides would greatly increase the percentage of labels that can be deciphered.

### Practical limitation 3: the dorsal view does not show enough characters

We argue that in many cases, images of drawers, usually showing dorsal view of specimens, will provide a very good first impression of what species look like and how credible a preliminary identification is. Dorsal views do provide ample feedback for the amateur, and the images will help researchers to better plan their research visits to remote museums (since they have a working knowledge of the contents of drawers before they leave their home laboratory). A dorsal view cannot replace careful specimen examination, but technology that is available now can provide images of sufficient quality to illustrate, for example, the tarsal structure of beetles less than a centimeter long. High resolution images viewed on a high definition screen will reveal more detail of more specimens than the average collection user will capture during a visit. We base this evaluation on currently operational technology (see articles in [51]); 3D scanning approaches under development will strongly advance the possibilities, supplementing the dorsal view with partial lateral, posterior and anterior views [44].

With reduced resources for supporting collections, it is important to find the most cost-effective ways of managing collections. Whole drawer imaging is the fastest and least expensive way to undertake specimen-level digitization. We assume that the greatest value of drawer digitization lies in the novel accessibility of millions of unsorted accessions specimens, discussed above (see also [30]). Expert taxonomists will, for most groups, be able to readily decide which specimens they would like to receive as loans for further examination, ecologists, conservationists or citizen naturalists can request additional data where needed to enhance research. Tagging options (e.g. such as those in flickr.com) will facilitate remote online curation, and in some cases might mobilize citizen scientists to participate.

### Practical limitation 4: sustainability - images are outdated quickly

Database maintenance is a recurring issue, no matter what kind of data are kept. As specimens are curated, drawer content changes. However, we argue that this does not prohibit mass digitization *per se*. Firstly, curators will prioritize drawers with an extraordinarily good curation status combined with high species content. The content of such drawers tends to be rather ‘stable’. Secondly, the amount of hands-on curator time required for whole drawer digitization is decreasing with technological advances and improved work-flows, so it is increasingly feasible to update all drawers that have been modified by a curator. This could be facilitated by assigning unique numbers to drawers (e.g., a barcode or a QR code on the outside of the drawers for a scanner attached to the imaging device or inside to capture this code as part of the image), which would permit specialist collection management software to automatically update the image file in the database and tagging of the image as ‘new’ or ‘updated’.

Curators will also focus on drawers which are in most urgent need of basic first-pass sorting – their composition should ideally be highly unstable as experts further sort and improve curatorial standard. Thus, for these unsorted drawers, the hope would be for a much faster turnover, which would require image update whenever unidentified specimens were removed for identification. Despite this, the net benefit in terms of curatorial improvement is higher than the effort for re-capturing the drawer contents. Other possibilities exist for tracking specimens with associated GUIDs once they have been moved from one unit tray to another. Alternatively, each unit tray of a drawer in a collection can be uniquely identified, and this code/serial number/barcode can be included in each image; when a specimen is moved from one databased unit tray to another, the new location data will go along with the specimen.

We feel that presently only a fraction of collections is actively being curated at a rate where drawer images will be outdated on a routine basis. Most institutions, even large ones, lack curators for most taxonomic groups. This means that on average whole sections of a collection can be imaged and the drawers will likely go unchanged for years to come – often decades. In the process of digitizing these largely static portions of a given collection, the collection itself is catalogued via images, which are very useful for insurance purposes, as well as getting closer to answering that age old museum question: ‘how many specimens and species *do* we have?’. Counting the number of specimens in a drawer can readily be achieved using existing image analysis software like ImageJ [52], which can, for example, assign numbers to each specimen in a drawer.

## Conclusion

Recent technical developments allow the creation and dissemination of high-resolution, zoomable images of natural history collection objects. The availability of high-resolution imaging technology has, for the first time, the potential to enable a dramatic increase in the speed of collection digitization efforts, in particular against the background of large collection holdings and limited personnel resources. We propose to create a distributed, virtual global collection of natural history specimens and to provide a comprehensive and authoritative ‘metacollection’ that is readily accessible on the internet. Most promising areas of application are collections of numerous specimens that are deposited in standard-sized drawers, such as insects, mollusk shells, etc. The pooled information from many collections allows new approaches of curating virtual world collections of certain taxa and to infer a wide range of biodiversity related information, morphological data, behavioral data, habitat type, geography, host plants, and ecological data. Limitations of the method need to be addressed, some of which are inherent to the method, such as labels that are hidden under the specimen, or inadequate, insufficient, or cryptic label data that require well-trained staff no matter what digitization methods are used. The amount of information that can be inferred from images that show specimens viewed only from above varies across taxa, but often provides enough feedback to make decisions regarding further research directions, the requirement to physically visit a collection, or which material needs to be borrowed for closer examination. Lastly, images may become outdated when the drawer content changes. However this is a general problem of any digitization effort and requires an efficient mechanism to update the information, in this case the ability to quickly and efficiently rescan a collection unit like an insect drawer. In addition, this will create a series of snapshots of a drawer that can serve as a timeline for the content of a particular collection unit or even for tracking individual specimens if these can be recognized in dorsal view by their ID or perhaps their barcode or QR code. A range of existing initiatives (e.g. GBIF, EoL, ALA) contain technical solutions which indicate that the creation of a global virtual collection Is not only possible, but that it will greatly facilitate our ways of understanding biodiversity by utilizing the enormous potential that is slumbering in our natural history collections worldwide.

## Competing interests

The authors declare that they have no competing interests.

## Authors’ contributions

MB and SS designed the study. All authors participated in manuscript preparation; all have read and approved the final manuscript.
